# Fructose Consumption Affects Glucocorticoid Signaling in the Liver of Young Female Rats

**DOI:** 10.3390/nu12113470

**Published:** 2020-11-12

**Authors:** Ivana Elaković, Sanja Kovačević, Danijela Vojnović Milutinović, Aleksandra Nikolić-Kokić, Alhadi M. Glban, Mihajlo Spasić, Luc Tappy, Ana Djordjevic, Gordana Matić, Jelena Brkljačić

**Affiliations:** 1 Department of Biochemistry, Institute for Biological Research “Siniša Stanković”, National Institute of Republic of Serbia, University of Belgrade, 142 Despot Stefan Blvd, 11060 Belgrade, Serbia; ivana.elakovic@gmail.com (I.E.); sanja.kovacevic@ibiss.bg.ac.rs (S.K.); dvojnovic@ibiss.bg.ac.rs (D.V.M.); hadimj2000@yahoo.com (A.M.G.); djordjevica@ibiss.bg.ac.rs (A.D.); gormatic@ibiss.bg.ac.rs (G.M.); 2 Department of Physiology, Institute for Biological Research “Siniša Stanković”, National Institute of Republic of Serbia, University of Belgrade, 142 Despot Stefan Blvd, 11060 Belgrade, Serbia; san@ibiss.bg.ac.rs (A.N.-K.); spasa@ibiss.bg.ac.rs (M.S.); 3 Department of Physiology, University of Lausanne, UNIL-CHUV, Rue du Bugnon 7, CH-1005 Lausanne, Switzerland; fructose1957@gmail.com

**Keywords:** glucocorticoid receptor, inflammation, antioxidant enzymes, lipin-1, lipogenesis, fructose-fed rat

## Abstract

The effects of early-life fructose consumption on hepatic signaling pathways and their relation to the development of metabolic disorders in later life are not fully understood. To investigate whether fructose overconsumption at a young age induces alterations in glucocorticoid signaling that might contribute to development of metabolic disturbances, we analysed glucocorticoid receptor hormone-binding parameters and expression of its target genes involved in gluconeogenesis (phosphoenolpyruvate carboxykinase and glucose-6-phosphatase) and lipid metabolism (lipin-1), as well as redox and inflammatory status in the liver of female rats subjected to a fructose-rich diet immediately after weaning. The fructose diet increased hepatic corticosterone concentration, 11β-hydroxysteroid dehydrogenase type 1 level, glucocorticoid receptor protein level and hormone-binding activity, as well as lipin-1 level. The expression of glucose-6-phosphatase was reduced in fructose-fed rats, while phosphoenolpyruvate carboxykinase remained unaltered. The fructose-rich diet increased the level of fructose transporter GLUT2, while the expression of fructolytic enzymes fructokinase and aldolase B remained unaltered. The diet also affected pro-inflammatory pathways, but had no effect on the antioxidant defence system. In conclusion, a fructose-rich diet applied immediately after weaning promoted lipogenesis and enhanced hepatic glucocorticoid signaling, possibly to protect against inflammatory damage, but without an effect on gluconeogenesis and antioxidant enzymes. Yet, prolonged treatment might ultimately lead to more pronounced metabolic disturbances.

## 1. Introduction

The modern way of living, which combines a sedentary lifestyle and decreased physical activity with overconsumption of highly palatable, energy-dense foods, is likely to contribute to modern health threats. Since the introduction of high-fructose corn syrup in the 1970s, dietary fructose daily intake has been largely increased. This was parallel with a rising prevalence of various metabolic disorders including obesity, metabolic syndrome, and type 2 diabetes [1,2]. Fructose is a lipogenic sugar, which can promote profound metabolic alterations in the liver, by deregulation of several key signaling and metabolic pathways [3].

Glucocorticoid hormones play an important role in the regulation of glucose and lipid metabolism [4]. Pathologically increased glucocorticoids, as in Cushing’s disease or after prolonged systemic glucocorticoid therapy, can lead to metabolic disorders including central obesity, ectopic lipid accumulation, hyperglycaemia, and so on, suggesting that disturbed glucocorticoid action may play an important role in the pathophysiology of obesity and metabolic syndrome [5,6]. Glucocorticoid secretion is mainly determined by the activity of the hypothalamic–pituitary–adrenal axis, however, their tissue levels are crucially dependent on the microsomal enzyme 11β-hydroxysteroid dehydrogenase type 1 (11βHSD1), which acts primarily as an oxido-reductase and catalysis in the production of active glucocorticoids (corticosterone in rats) from inactive 11-keto steroids (11-dehydrocorticosterone). In the tissue, glucocorticoids exert their effects by binding to glucocorticoid receptor (GR), a transcription factor activated by ligand binding [7]. Upon hormone binding, GR translocates from the cytoplasm to the nucleus, associates with glucocorticoid response elements (GRE) on DNA, and modulates transcription of target genes. Additionally, GR can interact with other transcription factors, including nuclear factor kappa B (NFkB), and indirectly regulate gene transcription [7].

In the liver, glucocorticoids modulate the expression of enzymes involved in lipid and glucose metabolism [8,9]. GR positively regulates expression of unique gluconeogenic enzymes in the liver, including rate-controlling enzyme phosphoenolpyruvate carboxykinase (PEPCK) [10] and glucose-6-phosphatase (G6P) [11], as well as lipogenic genes fatty acid synthase (FAS) and acetyl-CoA carboxylase (ACC) [8]. GRE sequences are also detected in the lipin-1 gene [8]. Lipin-1 modulates lipid metabolism through two distinct functions: acting as an enzyme phosphatidate phosphatase type 1 (PAP1), thus participating in triglyceride synthesis, or as transcriptional coregulator that regulates expression of genes involved in mitochondrial fatty acid oxidation [12]. Beside stimulation of lipin-1 gene expression, glucocorticoids increase PAP1 activity in the hepatocytes [13]. Apart from their metabolic role, glucocorticoids are involved in the regulation of inflammatory response, primarily by antagonizing proinflammatory transcription factors such as NFkB [14]. This effect may be relevant to metabolic disorders, as fructose-induced metabolic alterations are often accompanied by chronic low-grade inflammation [15,16,17].

Previous studies revealed the link between nutritional excess and oxidative stress, suggesting that redox disbalance might participate in the progression of metabolic disturbances including insulin resistance-related disorders [18,19,20]. Glucocorticoid signaling is also subject to redox-regulation [21]. Namely, GR contains 20 cysteine residues in its amino-acid sequence, five of them in the steroid binding domain [22], and the reduced state of the receptor sulfhydryl groups is considered a necessary prerequisite for its functioning [23,24]. However, the relation between fructose consumption and oxidative stress is rather complex, as fructose has been shown to produce both pro- and anti-oxidative effects, depending on the amount consumed, duration of consumption, and (patho)physiological milieu [25].

The young human population consumes more fructose-enriched soft drinks and juice beverages than adults, and has an increased risk of developing metabolic disorders [26,27]. Sex-related differences in susceptibility and progression of metabolic disorders have gained much attention recently. We have previously shown that young male and female rats employ different strategies to cope with dietary fructose-related energy overload in order to avoid lipid accumulation in the liver [28]. Furthermore, we have reported sex-specific differences in glucocorticoid signaling and expression of GR regulated genes involved in lipolysis and lipogenesis in the visceral adipose tissue of fructose-fed rats. Namely, long-term fructose consumption reduced GR expression and hormone binding potential, and altered expression of GR-target genes in visceral adipose tissue of female rats, implying that inability of GR to elicit lipolytic actions might contribute to the fructose-induced adiposity [29]. In contrast to females, a fructose-rich diet enhanced glucocorticoid signaling in visceral adipose tissue of male rats. The absence of fructose-related adiposity in males might be related to stimulated lipolysis rather than lipogenesis [30]. Moreover, another study from our group performed on males has shown that dietary fructose may perturb hepatic prereceptor glucocorticoid metabolism and lipogenesis, resulting in hypertriglyceridemia and attenuated hepatic insulin sensitivity [31]. Bearing in mind the observed sex differences in GR signaling in the visceral adipose tissue, and the role of glucocorticoids in hepatic lipid metabolism in males, we were prompted to analyse their role in hepatic metabolism upon fructose feeding in females, with the idea to provide additional insight into gender specificity in the development of metabolic disorders. In addition to sexual dimorphism in glucocorticoid signaling, sex-differences in inflammatory response were also reported [32,33,34], thus underlying the need for gender-specific studies. However, even within the same gender, the young organism and the adult one differ by their metabolic, hormonal, and physiological profiles. The link between increased fructose consumption in childhood and the development of metabolic disorders in adulthood is not fully elucidated, and studies on females are still scarce.

In this study, we tested the hypothesis that fructose overconsumption at a young age induces alterations in glucocorticoid signaling that might contribute to fructose-induced metabolic disturbances in the liver of female rats. To that end, the effects of a fructose-rich diet on circulating and hepatic corticosterone concentrations, GR hormone binding parameters, and intracellular distribution and expression of GR and its target genes involved in gluconeogenesis and lipid metabolism were measured in the liver of female rats subjected to fructose-rich diet in a period from weaning to adulthood. Furthermore, we examined the effects of a 9-week fructose-rich diet on hepatic redox settings and inflammatory status in terms of activity and expression of antioxidant enzymes: cytoplasmic copper-zinc superoxide dismutase (SOD1), mitochondrial manganese superoxide dismutase (SOD2), catalase (CAT), glutathione peroxidase (GSH-Px) and glutathione reductase (GSH-Red), and markers of lipid peroxidation, as well as the level of proinflammatory cytokines and NFkB intracellular distribution.

## 2. Materials and Methods

### 2.1. Animals and Treatment

Female Wistar rats (21 days old), were randomly divided into two experimental groups: control group—fed with commercial standard chow and drinking water, and fructose group—fed with the same chow and 10% (*w*/*v*) fructose solution instead of drinking water (*n* = 9 animals per group). The detailed chow composition was described previously [35]. Animals in both experimental groups had ad libitum access to food and drinking fluid during 9 weeks. Animals were kept under standard conditions, 22 °C with a 12 h light/dark cycle. All animal procedures were in compliance with the EEC Directive 2010/63/EU on the protection of animals used for experimental and other scientific purposes, and were approved by the Ethical Committee for the Use of Laboratory Animals of the Institute for Biological Research “Siniša Stanković”, University of Belgrade (No. 02-20/10).

### 2.2. Determination of Corticosterone in Plasma and Liver

After overnight fasting involving replacement of fructose solution with tap water, animals were sacrificed by rapid decapitation. Trunk blood was rapidly collected into EDTA-containing tubes and agitated slowly. Blood plasma was isolated by centrifugation at 1600× *g* for 10 min at room temperature and then stored at −80 °C for subsequent processing. Plasma corticosterone concentrations were measured using a Corticosterone Enzyme Immunoassay (EIA) kit according to the manufacturer’s instructions (Immunodiagnostic Systems LTD, The Boldons, UK). Absorbance at 450 nm (reference 650 nm) was measured spectrophotometrically (Multiskan Spectrum, Thermo Fisher Scientific, Waltham, MA, USA). Corticosterone concentrations were determined using the 4PL curve fitting method (Prism 5.0, GraphPad Software, Inc., La Jolla, CA, USA) and given as ng/mL. The assay sensitivity was 0.17 ng/mL. Intra-assay and inter-assay coefficients of variability were 5.9% and 8.9%, respectively.

For measurement of the tissue corticosterone level, cytoplasmic fractions of the liver of control and fructose-fed rats were used. Corticosterone levels were determined using the High Sensitivity EIA kit (Immunodiagnostic Systems LTD, The Boldons, UK) according to the manufacturer’s instructions. The plates were read at 450 nm and 650 nm on the Multiskan Spectrum (Thermo Fisher Scientific, Waltham, MA, USA). Corticosterone concentrations were determined using the 4PL curve fitting method and given as ng/mg of protein.

### 2.3. Tissue Preparations

After blood collection, livers were perfused with cold 0.9% NaCl and quickly excised, weighed, and stored in liquid nitrogen until use. After thawing, the tissue was homogenized using Janke–Kunkel Ultra Turrax in ice cold homogenization buffer (20 mM Tris-HCl, pH 7.4, containing 10% glycerol, 50 mM NaCl, 2 mM dithiothreitol, 1 mM EDTA-Na_2_, 1 mM EGTA-Na_2_, 20 mM Na_2_MoO_4_, protease, and phosphatase inhibitors). Homogenized tissue was centrifuged for 10 min at 2000× *g*, 4 °C, after which the supernatant (S1) was used to obtain cytosols, while pellet (P1) was used for the preparation of nucleosols. Supernatant S1 was centrifuged for 30 min at 20,000× *g*, 4 °C, to remove mitochondria, followed by 1 h at 105,000× *g*, 4 °C. The final supernatants were used as the cytosols, while pellets (microsomal fraction) were washed with 100 mM Na_4_P_2_O_7_, pH 7.4 (1 h at 105,000× *g*, 4 C) and resuspended in phosphate buffer (50 mM K_2_HPO_4_, 10 mM KH_2_PO_4_, 0.1 mM EDTA, 20% glycerol, and 0.1 mM dithiothreitol (DTT), pH 7.4). To obtain nucleosols, pellets P1 were washed in 0.5 mL homogenization buffer (10 min at 2000× *g*, 4 °C), resuspended in 1 vol (*w*/*v*) of NUN buffer (25 mM HEPES, pH 7.6, 1 M Urea, 300 mM NaCl, 1% Nonidet P-40, 2 mM DTT, 20 mM Na_2_MoO_4_, protease, and phosphatase inhibitors), and incubated for 1 h in ice bath with frequent vortexing. After incubation, samples were centrifuged (10 min at 8000× *g*, 4 °C) and the supernatants were used as the nucleosol.

For preparation of whole cell extracts, livers were homogenized in ice-cold RIPA buffer (50 M Tris-HCl pH 7.2, 1 mM EDTA-Na_2_, 150 mM NaCl, 0.1% sodium dodecyl sulphate (SDS), 1% Nonidet P-40, 0.5% sodium deoxycholate, 2 mM DTT, protease, and phosphatase inhibitors). The homogenates were sonicated (3 × 5 s, 1 A, 50/60 Hz), incubated for 60 min on ice with continuous agitation and frequent vortexing, and centrifuged (16,000× *g*, 20 min, 4 °C). All steps were conducted at 0–4 °C and all samples were stored in liquid nitrogen.

### 2.4. Steroid Binding Analysis

Determination of GR equilibrium binding parameters was done by a radioligand-receptor binding assay. Aliquots of cytosols (50 μL) were incubated for 18 h at 0 °C with 5–80 nM [^3^H]dexamethasone (Amersham Pharmacia Biotech, UK; specific activity 1.3 TBq/mM) in the absence and in the presence of 100-fold molar excess of unlabelled dexamethasone to determine total and non-specific binding, respectively. The unbound steroid was removed by incubation (10 min, on ice) with dextran-charcoal suspension. Samples were directly introduced into 3 mL of scintillation cocktail and counted in Rackbeta liquid scintillation counter (LKB) at a [^3^H] counting efficiency of ≈48%. Specific binding was calculated by subtracting non-specific from total binding. All measurements were performed in triplicates. The maximal number of GR binding sites (B_max_) and the apparent equilibrium dissociation constant (K_d_) were calculated using a software for fitting the saturation curves. The GR hormone binding potential was calculated as the B_max_/K_d_ ratio.

The reduction of reversibly oxidized glucocorticoid receptor cysteine residues was performed using DTT as a reducing agent. Cytosols were incubated (1 h, on ice) with 10 mM DTT prior to the steroid binding assay. Pre-treated cytosols were subsequently incubated (18 h on ice) with 80 nM [^3^H]dexamethasone in the absence and in the presence of 100-fold excess of the unlabelled dexamethasone. The removal of unbound steroid and radioactivity measurements were done as described above.

### 2.5. Antioxidant Enzymes Activity and General Redox State Parameters

For determination of antioxidant enzymes activity, 1 g of frozen livers from individual animals was homogenized in 10 vol. (*w*/*v*) of buffer (50 mM Tris, 0.25 M sucrose, 0.1 mM EDTA, pH 7.4) and sonicated (3 × 10 s at 10 MHz on ice) prior to 60 min centrifugation at 105,000× *g*. Final supernatants were stored in liquid nitrogen until use. The activities of SOD1, SOD2, CAT, GSH-Px, and GSH-Red were determined as previously described [36]. Thiol content was determined after incubation (20 min, 37 °C) of supernatants with 5 mM 5,5-dithiobis-2-nitrobenzoic acid. The absorbance was measured at 412 nm (ε_412_ = 13,600 M^−1^ cm^−1^). Total thiol content was expressed in μmol per mg of proteins.

For measurement of total glutathione (GSH), the supernatants obtained after homogenization were deproteinized by 5-sulfosalicylic acid (10% *w*/*v*). The level of both reduced and oxidized glutathione (herein referred to as total GSH) was determined by modified glutathione reductase–5,5′-dithio- bis (2-nitrobenzoic acid) recycling assay [37] and expressed as nmol GSH/mg protein. The level of lipid peroxidation was estimated by measurement of thiobarbituric acid reactive substances (TBARS) in the liver cell extracts prepared in the Tris buffer without sucrose, as described previously [36].

### 2.6. SDS-Polyacrylamide Gel Electrophoresis (SDS-PAGE) and Immunoblotting

After boiling in Laemmli’s sample buffer, proteins were resolved on 7% or 12% SDS-polyacrylamide gels, and transferred to the PVDF membrane. Membranes were incubated with a primary antibody. GR was detected using an anti-GR antibody (PA1 511A) (Affinity BioReagents, Golden, CO, USA). SOD1, SOD2, CAT, GSH-Red, GSH-Px, GLUT2, and 11βHSD1 were detected using Abcam (Abcam, Cambridge, UK) antibodies (ab13498, ab13533, ab16731, ab16801, ab22604, ab54460, and ab393364, respectively). β-actin was detected by AC-15 antibody (Sigma-Aldrich, St. Louis, MO, USA). NF-κB-p65 subunit, lipin-1, and lamin B were detected using antibodies from Santa Cruz Biotechnology (Santa Cruz Biotechnology, Dallas, TX, USA) (sc-372, sc-98450, and sc-6217, respectively). After being incubated with alkaline phosphatase linked secondary antibody (Amersham Pharmacia Biotech, Little Chalfont, UK), immunoreactive proteins were visualized by the enhanced chemifluorescence method (Amersham Biosciences). Quantitative analysis of immunoreactive bands was done using ImageQuant software. β-actin was used as equal load controls for whole cell extracts and cytosols, and lamin B for nucleosols.

### 2.7. RNA Isolation, Reverse Transcription, and Real-Time PCR

Total RNA was isolated from the liver of each animal using TRIzol^®^ Reagent (AmBion, Life Technologies, Carlsbad, CA, USA) and DNA contamination was removed by DNAse I treatment. cDNA was synthesized from 2 µg of RNA. Reverse transcription was performed using High-Capacity cDNA Reverse Transcription Kit (Applied Biosystems, Foster City, CA, USA) according to the manufacturer’s instructions, and cDNA was stored at −80 °C until use.

Quantification of PEPCK, TNFα, and IL6 gene expression was performed by TaqMan^®^ Real Time PCR method using ABI Prism 7000 Sequence Detection System. TaqMan^®^ Gene Expression primers and probes for PEPCK (Rn01529014_m1), TNFα (Rn01525859_g1), IL6 (Rn01410330_m1), and hypoxanthine phosphoribosyltransferase 1 (HPRT1) (Rn01527840_m1) were obtained from Applied Biosystems (Applied Biosystems, Foster City, CA, USA). The expression of G6Pase, fructokinase, and aldolase B was analyzed using Power SYBR^®^ Green PCR Master Mix (Applied Biosystems, Foster City, CA, USA), and specific primer (Metabion, Planegg, Germany) pairs for the following: G6Pase: F: 5′-GACCTCAGGAACGCCTTCTATG-3′, R: 5′-AGGAGATTGATGCCCACAGTCT-3′; fructokinase: F: 5′-ACGGATCGCAGGTGCCTAT-3′, R: 5′-AGCACAGTGCAGGAGTTGGA-3′; aldolase B: F: 5′-GCCACCTCACACAGCTTCTG-3′, R: 5′-TCGGTGAGCCATGATGACA-3′; and β-actin: F: 5′-GACCCAGATCATGTTTGAGAC-3′, R: 5′-AGGCATACAGGGACAACACA-3′, were used. Real-time PCR reaction was performed using QuantStudio™ Real-Time PCR Systems (Applied Biosystems, Foster City, CA, USA). The specificity of SYBR^®^ Green reaction was verified by melt curve analyses. No template control was included for each target gene to detect possible reagent contamination. Relative quantification of target mRNA was performed using the comparative 2^−ΔΔCt^ method [38]. The obtained results were analysed by Sequence Detection Software version 1.2.3 for 7000 System SDS Software RQ Study Application with a confidence level of 95% (*p* ≤ 0.05).

### 2.8. Data Presentation and Analysis

Each assay was performed in triplicate for each sample. Data are presented as means ± SEM. Unpaired Student’s *t*-test was used to compare differences between experimental groups. A probability level of *p* < 0.05 was considered statistically significant.

## 3. Results

### 3.1. Effects of Fructose-Rich Diet on Plasma and Liver Corticosterone Concentrations, and the Level of Hepatic 11βHSD1

**Figure 1:**
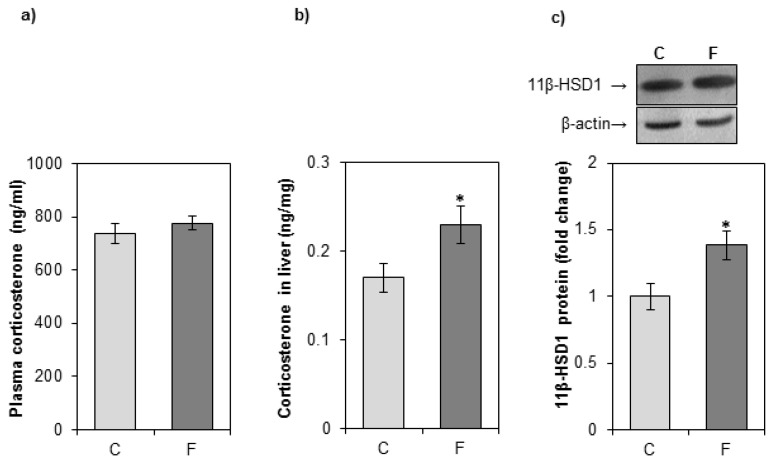
The effects of a fructose-rich diet on plasma and liver corticosterone concentrations, and the level of hepatic 11βHSD1. Groups: control (C), fructose-fed (F). Corticosterone concentrations in plasma (**a**) and liver (**b**) were measured by EIA kit. 11βHSD1 protein level in hepatic microsomal fraction (**c**) was measured by Western blot. Relative integrated optical density of the immunoreactive bands was assessed by ImageQuant software, normalized to β-actin, and expressed as fold of the control. The values represent the means ± SEM (*n* = 9). Statistical significance of differences between experimental groups: * *p* < 0.05.

As shown in Figure 1a, there was no significant change in the plasma corticosterone concentration between fructose-fed rats and rats on a standard diet. To evaluate the effect of fructose on the intracellular concentration of glucocorticoids, the hormone level was determined in cytoplasmic liver extracts. Corticosterone concentration in the liver of fructose-fed rats was increased as compared with control rats (Figure 1b). The protein level of 11βHSD1, the enzyme involved in local generation of active glucocorticoids, was increased in the liver of fructose-fed rats as compared with controls (Figure 1c).

### 3.2. Effects of Fructose-Rich Diet on Hepatic Glucocorticoid Receptor Signaling and the Level of Its Regulated Genes—Pepck, G6pase, and Lipin-1

**Figure 2:**
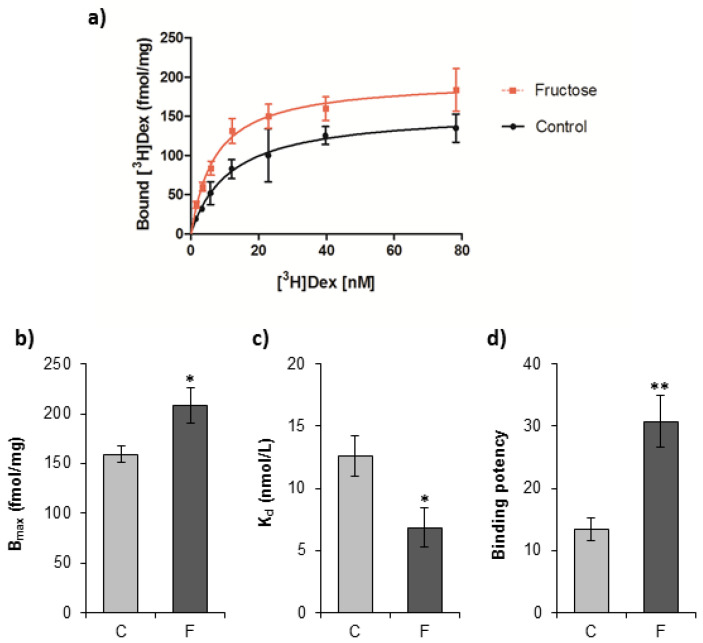
In vitro dexamethasone binding to glucocorticoid receptor (GR) from the liver of female rats subjected to a fructose-rich diet. Groups: control (C), fructose-fed (F). To measure dexamethasone binding to GR, aliquots of cytosols were incubated (18 h, 0 C) with increasing concentrations of [^3^H]dexamethasone (1–80 nM) in the presence and absence of 100-fold molar excess of unlabeled dexamethasone. Unbound [^3^H]dexamethasone was removed by dextran-charcoal. The saturation curves (**a**) were fitted to determine (**b**) the number of receptor binding sites (B_max_) and (**c**) equilibrium dissociation constant (K_d_). Relative binding potential (**d**) was calculated as the B_max_/K_d_ ratio. The values represent the mean ± SEM (*n* = 9). All measurements were done in triplicate. Statistical significance of differences between experimental groups: * *p* < 0.05; ** *p* < 0.01.

To investigate the effects of a fructose-rich diet on the GR hormone-binding function, the equilibrium binding parameters of [^3^H]dexamethasone interaction with GR as well as GR binding potential (B_max_/K_d_ ratio) were determined (Figure 2). GR hormone binding capacity (B_max_) and its affinity for the hormone (1/K_d_) were increased in fructose-fed rats as compared with controls (Figure 2b,c). Concomitantly, the GR hormone-binding potential was increased in the fructose-fed group as compared with controls (Figure 2d).

**Figure 3:**
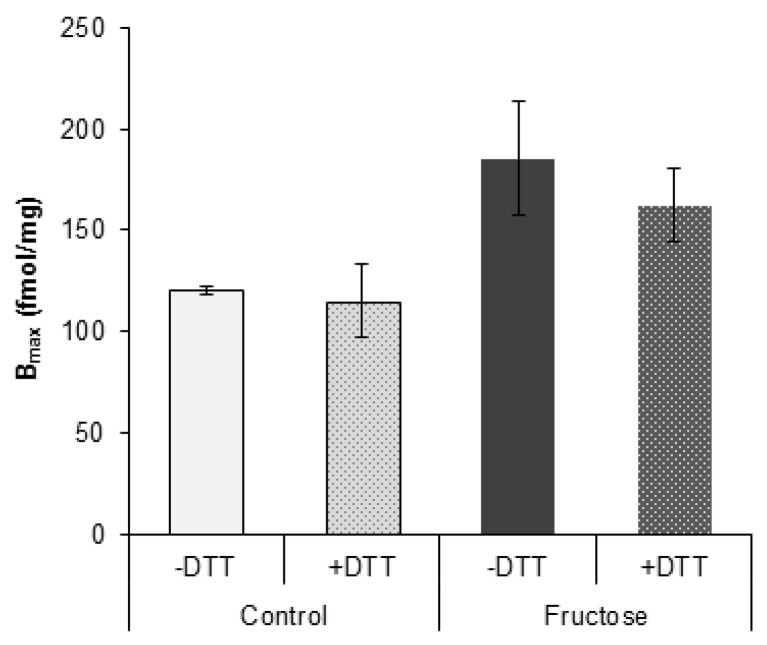
In vitro dexamethasone binding to hepatic GR in the presence and absence of thioprotector. Groups: control (C), fructose-fed (F). Cytosols were incubated (1 h, on ice) in the absence or in the presence of 10 mM DTT and subsequently incubated (18 h, on ice) with 80 nM [^3^H]dexamethasone in the absence and in the presence of 100-fold excess of the radioinert dexamethasone. Unbound [^3^H]dexamethasone was removed by dextran-charcoal and specific binding was calculated by subtracting non-specific from total binding. The values represent the mean ± SEM (*n* = 9). All measurements were done in triplicate.

To investigate possible oxidative modifications of GR, we compared GR hormone binding capacity in the absence and in the presence of reducing agent DTT. There were no significant changes in hormone binding capacity between DTT-treated and untreated samples within control or fructose group (Figure 3).

**Figure 4:**
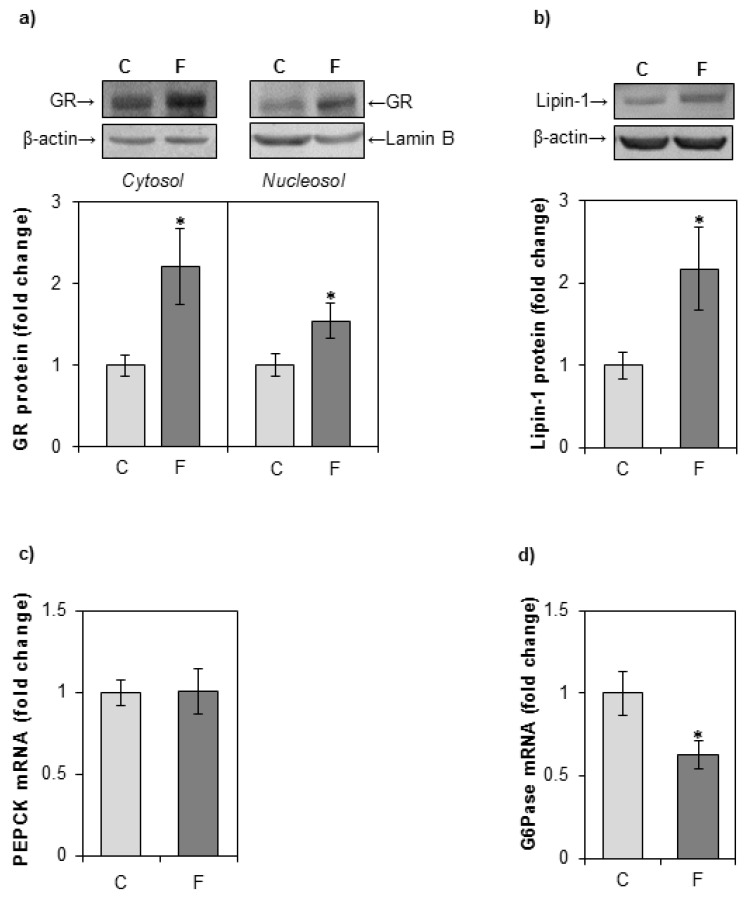
The effects of a fructose-rich diet on the level of GR, lipin-1 PEPCK, and G6Pase in the liver. Groups: control (C), fructose-fed (F). (**a**) GR protein level in hepatic cytosols and nucleosols, and (**b**) lipin-1 protein level in hepatic whole cell extracts, were measured by Western blot. Relative integrated optical density of the immunoreactive bands was assessed by ImageQuant software, normalized to equal load controls and expressed as fold of the control. The levels of PEPCK (**c**) and G6Pase (**d**) mRNA were determined by qPCR and expressed as fold of the control. The values represent the means ± SEM (*n* = 9). Statistical significance of differences between experimental groups: * *p* < 0.05.

GR protein levels in the cytoplasm and nuclei were determined by Western blotting. A fructose-enriched diet led to an increase in both cytosolic and nuclear GR protein level (Figure 4a). An increase in lipin-1 protein level was observed in whole cell extracts of fructose-fed rats as compared with controls (Figure 4b). Fructose overconsumption had no effect on the mRNA level of major gluconeogenic enzyme PEPCK (Figure 4c). The expression of G6Pase was decreased in fructose-fed rats as compared with rats on a standard diet (Figure 4d).

### 3.3. Effects of Fructose-Rich Diet on Hepatic Level of GLUT2, Fructokinase, and Aldolase B

**Figure 5:**
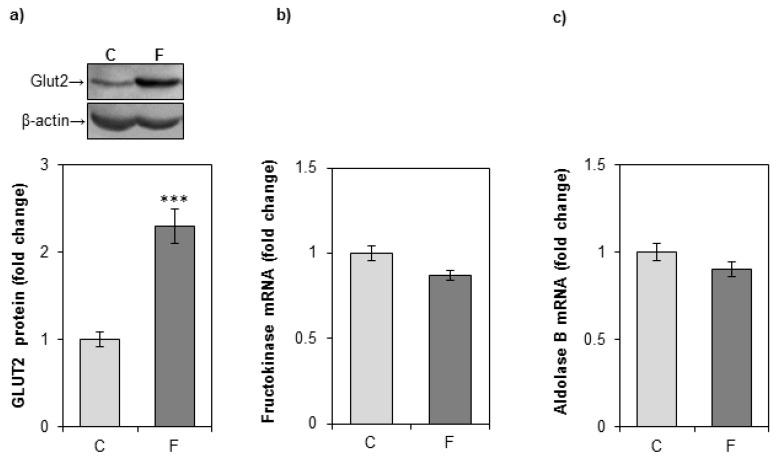
The effects of a fructose-rich diet on the level of GLUT2, fructokinase, and aldolase B in the liver. Groups: control (C), fructose-fed (F). (**a**) GLUT2 protein level in the hepatic whole cell extracts was measured by immunoblotting. Relative integrated optical density of the immunoreactive bands corresponding to GLUT2 was assessed by ImageQuant software, normalized to β-actin, and expressed as fold of the control. Representative Western blot is shown. The level of fructokinase (**b**) and aldolase B (**c**) mRNA relative to β-actin mRNA was determined by SYBR Green real-time PCR and expressed as fold of the control. The values represent the means ± SEM (*n* = 9). Statistical significance (Student’s *t*-test) of differences between experimental groups: *** *p* < 0.001.

A fructose-rich diet increased the protein level of fructose transporter GLUT2 in the liver, but had no effect on the expression of fructolytic enzymes fructokinase and aldolase B (Figure 5).

### 3.4. Effects of Fructose-Rich Diet on Hepatic Inflammatory and Redox Status

**Figure 6:**
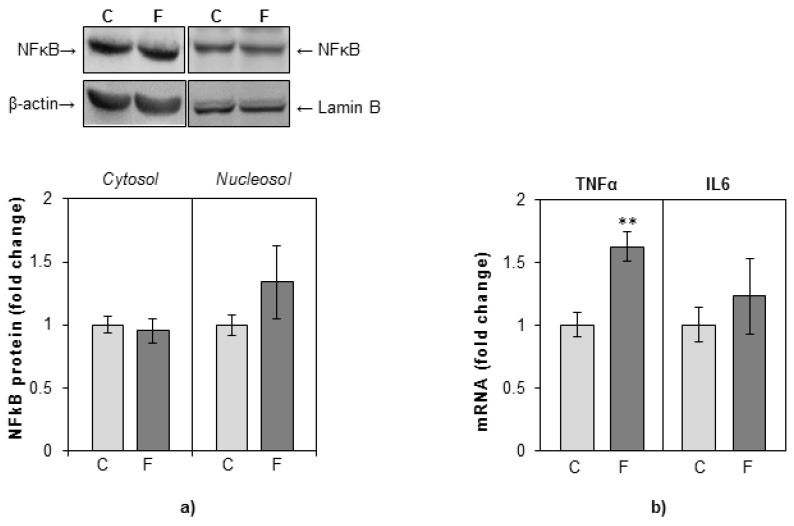
The effects of a fructose-rich diet on the level of NFkB, TNFα, and IL6 in the liver. Groups: control (C), fructose-fed (F). (**a**) NFkB protein level in hepatic cytosols and nucleosols was measured by Western blot. Relative integrated optical density of the immunoreactive bands corresponding to NFkB was assessed by ImageQuant software, normalized to equal load controls, and expressed as fold of the control. (**b**) The level of TNFα and IL6 mRNA relative to HPRT mRNA was determined by TaqMan real-time PCR and expressed as fold of the control. The values represent the means ± SEM (*n* = 9). Statistical significance of differences between experimental groups: ** *p* < 0.01.

The protein level of proinflammatory transcription factor NFκB and its intracellular redistribution between cytoplasm and nuclei were determined by Western blotting. There were no significant differences in the cytoplasmic and in the nuclear levels of NFκB between fructose-fed rats and rats on a standard diet (Figure 6a). The levels of proinflammatory cytokines TNFα and IL6 were determined by the real-time PCR method. An increase in TNFα gene expression was observed in fructose-fed rats as compared with controls, while the expression of IL6 gene remained unaltered (Figure 6b).

**Figure 7:**
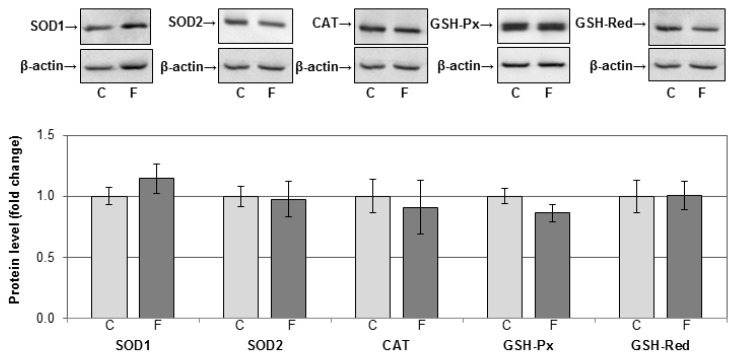
The effects of a fructose-rich diet on the level of antioxidant enzymes in the liver. Hepatic whole cell extracts (50 µg protein) were subjected to SDS-PAGE and Western blotting. Relative integrated optical density was assessed by ImageQuant software. β-actin was used as loading control. Representative Western blots and relative quantification of antioxidant enzyme protein levels of control (C) and fructose-fed rats (F) are shown. Values are means ± SEM (*n* = 9) and are presented as fold of the control. SOD1, cytoplasmic copper-zinc superoxide dismutase; SOD2, mitochondrial manganese superoxide dismutase: CAT, catalase; GSH-Px, glutathione peroxidase; GSH-Red, glutathione reductase.

To explore the possible pro-oxidative effects of fructose in hepatocytes, we determined the activity antioxidant enzymes SOD, CAT, GSH-Px, and GSH-Red in the rat liver. There was no significant difference in the activities of these enzymes between fructose-fed rats and rats on a standard diet (Table 1). The level of GSH, total thiols, and TBARS also remained unaltered (Table 1). The protein levels of SOD1, SOD2, CAT, GSH-Px, and GSH-Red were examined by Western blot and were not significantly different between fructose-fed rats and rats on a standard diet (Figure 7).

## 4. Discussion

Fructose present in the portal vein enters liver cells through GLUT2 or GLUT5 transporters to undergo rapid fructolysis by fructokinase and aldolase B. Fructose carbons can thereafter be processed through the tricarboxylic acid cycle, Cori cycle, pentose phosphate shunt, gluconeogenesis, and lipid synthesis pathways [39]. After chronic fructose consumption, a large portion of fructose carbons can be shifted to lipid synthesis, and an increased plasma triglycerides (TG) level is one of the most consistently reported effects of fructose overconsumption [17,39,40,41,42]. In line with these observations, we have previously reported that fructose overconsumption induced hypertriglyceridemia in the same young female rats, pointing out that the employed fructose diet regimen produces characteristic changes of metabolic syndrome [28]. In this study, we show that activation of hepatic GR might be one of the mechanisms that contribute to fructose-induced hypertriglyceridemia and disturbed lipid metabolism in young female rats.

Glucocorticoids are important regulators of glucose and lipid metabolism, and disturbed glucocorticoid action may be involved in the pathophysiology of various metabolic disorders. The results of this study show that a fructose-enriched diet enhances glucocorticoid signaling in the liver of young female rats. Although a 9-week long fructose-enriched diet had no effect on the plasma corticosterone level, this result is in agreement with previously published data showing that metabolic disturbances caused by fructose are not necessarily followed by changes in the systemic glucocorticoid level, but rather with intracellular hormone availability [29,30,43,44,45]. Indeed, in the present study, a fructose-rich diet elevated the hepatic 11βHSD1 level and intracellular corticosterone concentration, and this effect was accompanied by GR activation. An increase in hepatic GR hormone binding activity was evidenced by both an increased GR hormone binding capacity and an increased affinity for the hormone, which was reflected in a profound increase of overall receptor’s hormone-binding potential. An elevated number of hormone-binding sites, i.e., the maximal number of receptors that are capable to bind a hormone, can be attributed to the raised protein level of the GR. However, GR activation and its nuclear accumulation, observed in the liver of fructose-fed rats, were not followed by increased expression of the GRs’ target gene PEPCK. Previous studies have shown unaltered or even downregulated expression of PEPCK in fructose-fed rodents [16,41,46]. Using the same animal model, we have previously reported decreased plasma glucose level in fructose-fed female rats [47], which goes in line with the decreased expression of G6Pase observed herein. Nevertheless, systemic insulin resistance was not observed in this animal model, as confirmed by unaltered plasma insulin, HOMA index, and intraperitoneal glucose tolerance test [47]. As glucocorticoids exert stimulatory effects on gluconeogenesis in starvation and stress, it is possible to assume that, in our experimental setting, where increased caloric intake was observed [28], activated GR might rather modulate lipid metabolism than gluconeogenesis, possibly participating in channelling fructose carbons towards lipid synthesis. In order to definitely confirm such a role of GR, a follow-up study including treatment of the rats with a GR agonist is needed.

We observed an increase of the hepatic GLUT2 fructose transporter expression, which is consistent with an enhanced fructose entry into hepatocytes. The expression of fructolytic enzymes, fructokinase and aldolase B, remained unaltered. However, as these enzymes have high activity, and because aldolase B, unlike phosphofructokinase in the glycolytic pathway, is not inhibited by intracellular substrates, this remains consistent with an enhanced hepatic fructose metabolism [48,49]. In addition, a fructose-rich diet raised the protein level of lipin-1. Glucocorticoids were shown to increase hepatic lipin-1 mRNA level, protein level, and PAP1 activity both in vitro and in vivo [50,51]. Increased PAP1 activity enables the liver to divert fatty acids into TGs. The fructose-induced increase in the hepatic lipin-1 protein level, observed herein in the whole cell extract, most likely reflects enhanced PAP1 activity. This is based on our previous result showing an unchanged nuclear level of lipin-1 in the same animals [28]. This also goes in line with our finding that fructose stimulates de novo synthesis of free fatty acids in the liver of female rats (evidenced by increased expression of GR-target genes ACC1 and FAS), and increases the plasma TG level, rendering β-oxidation of fatty acids unaltered [28]. However, despite increased synthesis and elevated plasma level, TG content in the liver of fructose-fed female rats remained unaltered [28]. It has been proposed that adipocytes have the ability to accommodate the caloric surplus of overnutrition, thus minimizing ectopic lipid accumulation [52]. Indeed, using the same animals, we have previously shown that a fructose-rich diet induced the enlargement of visceral adipose tissue in young female rats [29]. However, the visceral adipose tissue can protect from ectopic lipid deposition only to the certain extent, after which its enlargement poses a greater risk for the development of metabolic disturbances [52]. It is possible to assume that prolonged fructose feeding could ultimately disrupt the ability of visceral adipose tissue to deal with large amounts of lipids, thus enabling ectopic lipid accumulation.

Lipids might serve as substrates in harmful chain reactions such as lipid peroxidation, thereby contributing to development and progression of metabolic disorders. The results of the current study show that a fructose-rich diet did not induce lipid peroxidation and oxidative stress in the liver. Namely, the diet had no effect on the protein level and activity of antioxidant enzymes. Moreover, the levels of GSH and free thiol groups as markers of general redox conditions, and the level of TBARS as a marker of lipid peroxidation, remained unaltered after fructose consumption. In addition, redox-related changes in GR activity were not observed, as evidenced by pre-treatment with reducing agent DTT. However, data from the literature suggest that fructose overconsumption can lead to disruption of antioxidant mechanisms in the liver of adult rats [53,54,55,56,57]. The large discrepancies in the course and intensity of fructose-induced alterations might be attributed to variations in treatment duration, the form (liquid or solid) and amount of fructose, as well as the sex and age of the animals at the beginning of treatment.

The efficiency of the antioxidant defence system is largely dependent on age, and we have previously reported the absence of oxidative stress in young male rats subjected to 9-week treatment with 10% or 60% fructose solution immediately after weaning [36,58]. The current results show that young female rats also have the capacity to maintain hepatic redox homeostasis when challenged by a fructose rich diet. However, using the same animals, we have previously shown that a fructose-rich diet alters antioxidant enzymes function in visceral adipose tissue [59]. These tissue-specific differences might be, at least in part, attributed to the superior capacity of the hepatic antioxidant system as compared with other tissues [60].

Fructose consumption was shown to induce low-grade inflammation in the liver [41,61]. In the current study, fructose led to an increase in TNFα expression, which was not accompanied by an increased level of proinflammatory cytokine IL6, nor by marked NFκB activation. Given that GR has the ability to transrepress the activity of NFκB, the observed GR activation in fructose-fed female rats could be instrumental in the suppression of inflammation. In support of this hypothesis, treatment with dexamethasone counteracted cholestasis-related hepatic inflammation by preventing NFκB translocation to the nucleus and restored TNFα, IL-6, and IL-1β mRNA levels in the liver of cholestatic rats [62]. In contrast to fructose-fed female rats, we have previously found that fructose activated NFκB and elevated TNFα expression in the liver of male rats subjected to fructose-rich diet after weaning, which was in concert with the absence of hepatic GR activation [31,46,63]. Sex differences in GR signaling might, at least in part, explain why young male, but not female rats exhibit NFκB activation and marked proinflammatory status in the liver when subjected to a fructose-rich dietary regime. It seems that genders may differ in the ability of corticosterone to suppress the progression of metabolic inflammation.

## 5. Conclusions

The results of this study show that a fructose-rich diet applied immediately after weaning in female rats enhances GR activity. GR activation might contribute to shifting fructose carbons towards lipogenesis, and could also protect against inflammatory damage. However, this was not followed by hepatic lipid accumulation or oxidative stress. We could assume that young female rats have the ability to preserve metabolic flexibility, maintain redox homeostasis, and modulate inflammatory response in order to avoid pathophysiological changes in the liver posed by fructose overconsumption. Nevertheless, it is possible to assume that prolonged treatment would ultimately lead to more pronounced metabolic disturbances in later adulthood.

## Figures and Tables

**Table 1:** Antioxidant enzyme activities and the level of glutathione (GSH), total thiols (SH), and thiobarbituric acid reactive substances (TBARS) in the liver of female rats subjected to a fructose-rich diet over a period from weaning to adulthood. SOD1, cytoplasmic copper-zinc superoxide dismutase; SOD2, mitochondrial manganese superoxide dismutase: CAT, catalase; GSH-Px, glutathione peroxidase; GSH-Red, glutathione reductase.

	Control	Fructose
SOD1 (U/mg protein)	26.13 ± 4.33	26.12 ± 4.45
SOD2 (U/mg protein)	7.42 ± 0.29	6.39 ± 0.46
CAT (U/mg protein)	275.71 ± 15.64	248.13 ± 13.25
GSH-Px (U/mg protein)	1113.9 ± 32.64	1082.91 ± 32.35
GSH-Red (U/mg protein)	63.14 ± 1.41	65.82 ± 1.37
GSH (nmol/mg protein)	0.145 ± 0.010	0.141 ± 0.007
SH groups (µM/mg protein)	0.052 ± 0.004	0.049 ± 0.006
TBARS (nmol/mg protein)	0.382 ± 0.022	0.336 ± 0.012

Comparisons between fructose-fed and control rats were made by unpaired Student’s *t*-test. Values are expressed as the mean ± SEM (*n* = 9).
